# A Novel Peptide Binding Prediction Approach for HLA-DR Molecule Based on Sequence and Structural Information

**DOI:** 10.1155/2016/3832176

**Published:** 2016-05-31

**Authors:** Zhao Li, Yilei Zhao, Gaofeng Pan, Jijun Tang, Fei Guo

**Affiliations:** ^1^School of Computer Science and Technology, Tianjin University, 92 Weijin Road, Nankai District, Tianjin 300072, China; ^2^School of Computational Science and Engineering, University of South Carolina, Columbia, SC, USA

## Abstract

MHC molecule plays a key role in immunology, and the molecule binding reaction with peptide is an important prerequisite for T cell immunity induced. MHC II molecules do not have conserved residues, so they appear as open grooves. As a consequence, this will increase the difficulty in predicting MHC II molecules binding peptides. In this paper, we aim to propose a novel prediction method for MHC II molecules binding peptides. First, we calculate sequence similarity and structural similarity between different MHC II molecules. Then, we reorder pseudosequences according to descending similarity values and use a weight calculation formula to calculate new pocket profiles. Finally, we use three scoring functions to predict binding cores and evaluate the accuracy of prediction to judge performance of each scoring function. In the experiment, we set a parameter α in the weight formula. By changing α value, we can observe different performances of each scoring function. We compare our method with the best function to some popular prediction methods and ultimately find that our method outperforms them in identifying binding cores of HLA-DR molecules.

## 1. Introduction

Histocompatibility refers to the degree of antigenic similarity between the tissues of different individuals, which determines the acceptance or rejection of allografts. Transplantation antigen or histocompatibility antigen is the cause of rejection of allografts [1, 2]. MHC (Major Histocompatibility Complex) is present on the chromosome encoding a major histocompatibility antigen, mutual recognition between control cells, and the regulation of immune response.

MHC molecule plays a key role in immunology, and the molecule binding reaction with peptide is an important prerequisite for T cell immunity induced [2, 3]. By detecting a wide variety of microbial pathogens, the immune system protects host against diseases. Because of this, the binding prediction of MHC molecules with peptides has always been a hot topic in bioinformatics. Many researches in this field not only help us to understand the process of immune but also develop the work of vaccine design assisted by computers.

MHC genes produce two different types of molecules, which are MHC I molecules and MHC II molecules [1, 2]. MHC I molecules contain two separate polypeptide chains: the MHC *α* chain encoded by MHC genes and the MHC *β* chain encoded by non-MHC genes [4, 5]. MHC I class molecules are expressed in almost all eukaryotic cell surfaces, recognized by CD8+ cells. MHC II class molecules consist of two non-covalently linked polypeptide chains, namely, *α* chain and *β* chain. MHC II class molecules are expressed on antigen-presenting cells in general. Foreign MHC II antigens only capture and present on the surface of antigen-presenting cells (APC) TH cell [6]. After that, APC secretes large amounts of cytoplasm, activating cell invasion defensed behavior. Only the binding of antigen peptides and MHC II class molecules can activate CD4+ TH cells (helper T cells) [7]. Then, the activated TH cells would differentiate into effector cells and activate the immune response.

The structures of MHC I molecules and MHC II molecules slightly differ in the binding grooves [5]. Close grooves form on the binding of MHC I molecules and antigenic peptides. On the other hand, MHC II molecules do not have conserved residues, so they appear as open grooves. As a consequence, this will increase the difficulty in predicting MHC II molecules binding peptides [7]. In this paper, we aim to solve more difficult problem of predicting MHC II binding peptides.

The pioneering and most popular pan-specific approach for MHC II binding prediction is the TEPITOPE method [8], and basic idea is the HLA-DR allele having identical pseudosequence. The same pocket will share the same quantitative profile. By using multiple instance learning, the MHCIIMulti method [9] can predict more than 500 HLA-DR molecules. Transforming each DRB allele into a pseudosequence with 21 amino acids and using the SMM-align method to identify binding cores, the NetMHCIIpan method [5] gets an accurate prediction by using an artificial neural network algorithm [10, 11]. Combining NN-align and NetMHCpan with NetMHCIIpan [9, 12], the MULTIPRED2 method [13–15] can get a perfect prediction for 1077 HLA-I and HLA-II alleles and 26 HLA supertypes.

In this paper, we propose a novel prediction method for predicting MHC II molecules binding peptides. First, we calculate sequence similarity and structural similarity between different MHC molecules [13, 16]. Then, we reorder pseudosequences according to descending similarity values and use a weight calculation formula to calculate new pocket profiles. Finally, we use three scoring functions to predict binding cores and evaluate the accuracy of prediction to judge performance of each scoring function [17, 18]. In the experiments, we set a parameter *α* in the weight formula. By changing *α*  value, we can observe different performances of each of the scoring functions. We compare our method with the best function to some popular prediction methods and ultimately find that our method outperforms them in identifying binding cores of HLA-DR molecule [19]. The work would suggest a novel computational strategy for special protein identification instead of traditional machine learning based methods [20, 21].

## 2. Materials and Methods

### 2.1. Data Sets

We find 39 MHC molecules and peptides binding complexes from Protein Data Bank (http://www.rcsb.org/pdb/search/), which constitutes the data set used in this paper. In this data set, lengths are between 11 and 23, and we can find polypeptide-binding sites, namely, binding cores.  Table 1 lists the details of these 39 MHC molecules and peptide binding complexes [14, 22, 23].

In Table 1, the first column is PDB ID of 39 complexes from PDB; the second column is the name of corresponding alleles from 39 complexes; the third column is the corresponding polypeptide sequences, in which the enlarged nine positions are the binding cores.

### 2.2. Methods

**Figure 1:**
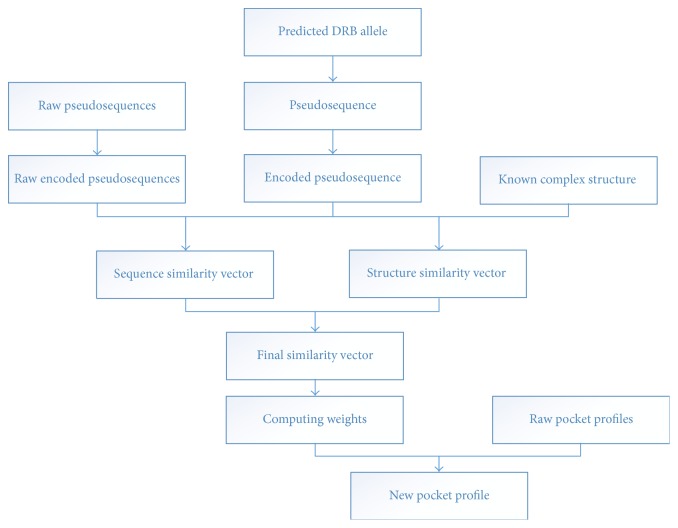
The architecture of our approach to MHC II and peptide binding problem.

There are thousands of allele variants in nature [2, 4]. It is absolutely impossible to measure the binding specificity one by one. Motivated by this perspective, we propose a new computational method to predict the binding specificity of peptides without any biochemical experiment, which combines the sequence and structural information of these known specificity-binding MHC molecules, as showed in Figure 1. We evaluate the method on all general HLA-DRB data sets, and results indicate that our method is close to the state-of-the-art technology and our approach can predict all sequence-known MHC molecules and cost little time, extending the prediction space compared with other time-consuming approaches.

### 2.3. Crucial Pockets relative to Binding Specificities of HLA-DR Molecules

We mainly use Position Specific Scoring Matrix (PSSM) [13, 24] in our approach, which is a popular technology in the problem of MHC binding. Roughly speaking, there are nine amino acids in MHC binding cores, and each position is a specific pocket as showed in Table 2. We use PSSM to quantify the binding affinity between twenty basic amino acids with these nine pockets.

There are five anchor sites (1, 4, 6, 7, and 9) at the binding core for MHC II molecules, which determine the binding strength of peptides with MHC II molecules. Because site 1 of MHC II is consistent with different MHC II molecules and peptides, it is important to identify the precise quantification of its binding core in site 1, yet we use weights of four anchor sites (4, 6, 7, and 9) to define profiles. For other sites, the same approach, such as TEPITOPE, is to specify their quantitative profiles.

### 2.4. Computing Similarity between Different MHC Molecules

#### 2.4.1. Sequence-Based Similarity

Sequence-based similarity can be calculated by alignment results. Here, pocket pseudosequences and associated profiles refer to raw pocket pseudosequences and raw pocket profiles, respectively. These raw pseudosequences are composed of several amino acids, whose associated residue indices are shown in Table 3. Eleven representative HLA-DR alleles are adopted to specify different profiles for anchor pockets 4, 6, 7, and 9. These eleven alleles are DRB1^*∗*^0101, DRB1^*∗*^0301, DRB1^*∗*^0401, DRB1^*∗*^0402, DRB1^*∗*^0404, DRB1^*∗*^0701, DRB1^*∗*^0801, DRB1^*∗*^1101, DRB1^*∗*^1302, DRB1^*∗*^1501, and DRB5^*∗*^0101. If two alleles have identical pseudosequences in the same pocket, they will have identical profiles. For a given pocket, we collect all the different raw pocket pseudosequences into one set *R* ^*x*^, *R* ^*x*^ = {*r*_1_, *r*_2_,…, *r*_*m*_}, and |*r*_*i*_| = *n*, where *i* = 1,2,…, *m*, *x* ∈ {4, 6, 7, 9}, *m* is the number of unique pseudosequences, and *n* is the number of amino acids contained in a pseudosequence. Meanwhile, we collect all different raw profiles into one set *P* ^*x*^, *P* ^*x*^ = {*p*_1_, *p*_2_,…, *p*_*m*_}, and |*p*_*i*_| = 20, where *i* = 1,2,…, *m*. There is a one-to-one correspondence between *p*_*i*_ and *r*_*i*_. We use BLOSUM to calculate the sequence similarity between different MHC molecules, defined as BLOSUM = (*S*_*q*_ − *S*_*i*_). Then, we can get encoded pseudosequence, which is a 20*n*-dimensional real vector *V* ^*x*^ = {*V*_1_, *V*_2_,…, *V*_*m*_}. We use Radial Basis Function (RBF) to measure the similarity between encoded predicted pseudosequences *V*_*a*_ and a raw encoded pseudosequence:

(1) $$\begin{array}{c} K_{seq}\left(\;V_{a},V_{i}\;\right)=\mathrm{BLOSUM}\left(\;V_{a},V_{i}\;\right),\;V_{i}\subseteq V^{x}. \end{array}$$

#### 2.4.2. Structure-Based Similarity

Using MHC II HLA-peptide complex structure from Protein Data Bank (PDB), we can get the residues 3D-coordinate of the pocket in each MHC molecule, *h* (*p*_*x*_, *p*_*y*_, *p*_*z*_). We define vector *H* ^*x*^ = {*h*_1_, *h*_2_,…, *h*_*n*_}, where *n* is the number of amino acids in the pseudocontained sequence; meanwhile, we collect a set *S* ^*x*^, *S* ^*x*^ = {*H*_1_, *H*_2_,…, *H*_*m*_}, *m* is the number of different pseudosequences, and there is also one-to-one correspondence between *H*_*i*_ and *r*_*i*_.

Next, we need to estimate the similarity of three-dimensional structures between a measured MHC molecule and five MHC molecules with known pseudosequence PSSM. Rigid transformation is to compare three-dimensional substructures of two proteins [25, 26].

Intuitively, we fix one of the structures, A, move (translation and rotation) the other structure, B, and find the best movement in three-dimensional space, with two atoms to the nearest structure. We calculate the Euclidean distance between two structures, defined as RMSD = |*C*_*q*_ − *C*_*i*_|. We can get encoded pseudosequence *V* ^*x*^ = {*V*_1_, *V*_2_,…, *V*_*m*_} and calculate the similarity between 3D structures of encoded predicted pseudosequences *V*_*a*_ and a raw encoded pseudosequence:

(2) $$\begin{array}{c} K_{spa}\left(\;V_{a},V_{i}\;\right)=\mathrm{RMSD}\left(\;V_{a},V_{i}\;\right),\;V_{i}\subseteq V^{x}. \end{array}$$

#### 2.4.3. Overall Similarity

After that, we have obtained sequence similarity and structural similarity. We calculate final similarity score functions according to the following three formulas:

(3) K1Va,Vi=KseqVa,Vi2+KspaVa,Vi22,K2Va,Vi=KseqVa,Vi+KspaVa,Vi2,K3Va,Vi=KseqVa,Vi+KspaVa,Vi.

### 2.5. Weights Calculation for New Pocket Profiles

We reorder all pseudosequences according to descending similarity values and use a weight calculation formula to calculate new pocket profiles. A new pocket profile is generated as a weighted average over *m* raw pocket profiles in *P* ^*x*^. Next, we use the gamma distribution to generate the weights. The gamma PDF distribution is defined as follows:

(4) $$\begin{array}{c} g\left(\;x;k,\theta \;\right)=\frac{1}{\theta^{k}}\frac{1}{\gamma \left(k\right)}x^{k-1}e^{-x/\theta }, \end{array}$$

where *x* > 0 and *k*, *θ* > 0, and *γ*(*k*) denotes the gamma function.

The weight distribution is generated to discretize the gamma PDF as follows:

(5) $$\begin{array}{c} G\left(\;X=i\;\right)=\frac{1}{\theta^{k}}\frac{1}{\gamma \left(k\right)}i^{k-1}e^{-i/\theta },\;i=1,2,\ldots ,m, \end{array}$$

where *m* is the dimension of the weights and *k* and *θ* are the shape and scale parameters, respectively. The gamma distribution generates the weight vector to give a higher weight for more similarity pseudosequences.

After normalizing, the weight vector is defined as follows:

(6) $$\begin{array}{c} P\left(\;X=i\;\right)=\frac{{\left[G\left(X=i\right)\right]}^{\alpha }}{\sum_{k=1}^{m}{\left[G\left(X=k\right)\right]}^{\alpha }},\;i=1,2,\ldots ,m. \end{array}$$

Given a predicted DRB allele *a*, let *K*_*a*_ = (*K*_*a*1_,*K*_*a*2_,…, *K*_*am*_), where *K*_*ai*_ = *K*(*V*_*a*_, *V*_*i*_), *V*_*i*_ ∈ *V* ^*x*^, and *α* is a positive number and enhances the weight vector to protect the outstanding contribution of most similarity pseudosequences. Associated raw pocket profiles are *P*_*x*_ = {*P*_1_, *P*_2_,…, *P*_*m*_}. Elements of *K*_*a*_ are sorted in descending order, and the reordered vector of *K*_*a*_ is denoted as $\tilde{K_{a}}=(\tilde{K_{a1}},\tilde{K_{a2}},\ldots ,\tilde{K_{am}})$. The corresponding weight vector is denoted as *W* = (*ω*_1_, *ω*_2_,…, *ω*_*m*_). We denote pocket profiles associated with the reordered vector $\tilde{K_{a}}$ as ${\tilde{P}}^{x}$, ${\tilde{P}}^{x}=\{{\tilde{P}}_{1},{\tilde{P}}_{2},\ldots ,{\tilde{P}}_{m}\}$. We define the pocket profile for allele *a* as follows:

(7) $$\begin{array}{c} {\tilde{P}}_{a}^{x}=\omega_{1}{\tilde{P}}_{1}+\omega_{2}{\tilde{P}}_{2}+\cdots +\omega_{m}{\tilde{P}}_{m}, \end{array}$$

where *x* ∈ {4,6, 7,9}.

## 3. Result

First, we design an experiment to choose appropriate scoring function to combine sequence similarity and structural similarity. Then, we compare with other state-of-the-art technologies, which are TEPITOPE, MultiRTA, NetMHCIIpan-2.0, and NetMHCIIpan-1.0. The result indicates that our approach can obtain better prediction and effectively extend current prediction methods. Finally, we test on more data sets.

### 3.1. Evaluation of Different Scoring Functions

**Figure 2:**
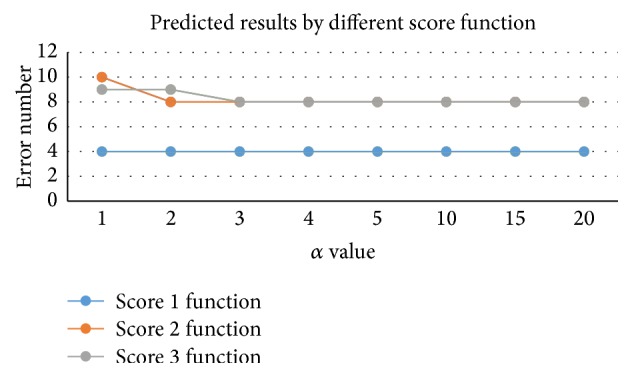
Predicted results by different score functions. *x*-axis represents different *α* values, and the *y*-axis refers to predicted results of different score functions.

Here, we use 30 of 39 MHC molecules and peptide complexes as test set and get the appropriate scoring functions as showed above. The value of the parameter *α* is set to 1, 2, 3, 4, 5, 10, 15, and 20, followed by results shown in Figure 2. We find that no significant changes can be found by *K*_1_(*V*_*a*_, *V*_*i*_); for *K*_2_(*V*_*a*_, *V*_*i*_) and *K*_3_(*V*_*a*_, *V*_*i*_), when *α* = 1 prediction error number is 10 and 9 and when *α* = 3 prediction errors reduced to 8, we set the value of *α* to 3. Comparing these three functions, the least numbers of errors by three functions are 4, 8, and 8. Details are shown in Tables S1, S2, and S3, in the Supplementary Material available online at http://dx.doi.org/10.1155/2016/3832176.

### 3.2. Compared with Conventional Well-Known Methods

From the above experimental results, *K*_1_(*V*_*a*_, *V*_*i*_) obtains the most accurate prediction, so we will select *K*_1_(*V*_*a*_, *V*_*i*_) with *α* = 3 as our final approach. We compare our current prediction results with conventional well-known methods TEPITOPE [23], MultiRTA [13], NetMHCIIpan-2.0 [12], and NetMHCIIpan-1.0 [12], and these results are shown in Table 4.

**Figure 3:**
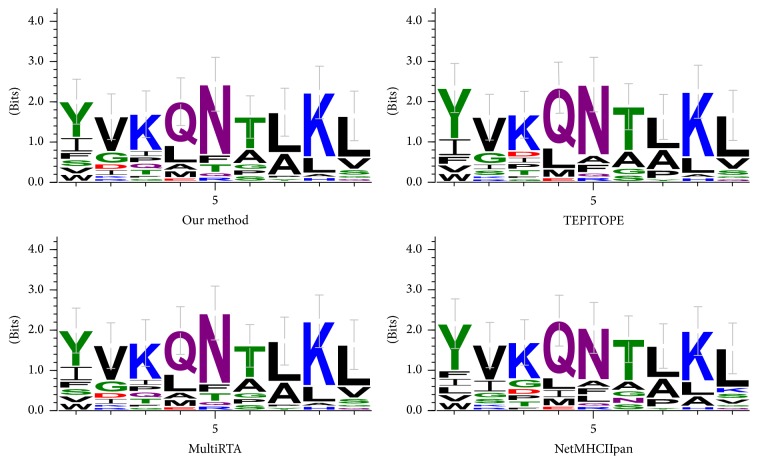
Comparison of different methods by sequence logos of peptides on HLA-DRB1^*∗*^0101.

TEPITOPE is a relatively early method and is one of the most popular methods for predicting MHC II binding molecules. The basic idea is that if two HLA-DR alleles have the same pseudorandom sequence in the same pocket, they share the same number of profiles. Through multiple instances, MHCIIMulti has predicted over 500 HLA-DR molecules. NetMHCIIpan firstly converts each of the DRB alleles into a pseudorandom sequence of 21 amino acids, then uses the SMM-align method to identify binding residues in the peptide chain and the core side, and finally uses artificial neural network to train the model. MultiRTA makes prediction on HLA-DR and HLA-DP molecules. By thermodynamic method, it calculates a peptide chain and all other residues to predict the average binding affinity of binding strength and the introduction of standardization constraints to avoid overfitting. MULTIPRED2 can predict 1077 HLA-I and HLA-II genes and 26 HLA supertypes. Details are as shown in Figure 3. Our method obtains 4 errors; however, TEPITOPE, MultiRTA, NetMHCIIpan-2.0, and NetMHCIIpan-1.0 get the numbers of errors as 0, 4, 6, and 3, respectively. Because now we only find five MHC II molecules with three-dimensional structural information, we use the scoring matrix with only 5 MHC II molecules. If the three-dimensional structural information of MHC II molecules can be extended to all of the 11 MHC II molecules, our predictions will be more accurate. From the current view, our approach has reached a higher level of prediction.

### 3.3. Other Prediction Results

When compared with other methods on the above experiments, we only use 30 of 39 MHC molecules and peptide complexes as test set. In this section, we test on the remaining nine MHC molecules. In this experiment, we choose *K*_1_(*V*_*a*_, *V*_*i*_) and set the parameter *α* = 3. As seen in Table 5, eight of nine predictions are accurate. Therefore, our approach produces a considerably great performance.

## 4. Conclusion

In this paper, we try to solve the problem of predicting MHC II binding peptides with a novel metric and strategy. Sequence similarity and structural similarity between different MHC molecules are calculated to reorder pseudosequences according to descending similarity, and then a weight calculation formula is used to calculate new pocket profiles. Finally, we use three scoring functions to predict binding cores and evaluate the accuracy of prediction to judge performance of each scoring function. In the experiment, we set a parameter *α* in the weight formula. By changing *α*  value, we can observe different performances of each scoring function. Then, we compare our method with the best function to some popular prediction methods and ultimately find that our method outperforms them in identifying binding cores of HLA-DR molecules.

## Supplementary Material

Using different functions to combine sequence similarity and structural similarity, these are the predicted results with the value of alpha ranging from 1 to 5.

## Figures and Tables

**Table 1:** Details of 39 MHC molecules and peptide binding complexes.

PDB ID	DRB allele	Peptide sequence
1AQD	DRB1^*∗*^0101	VGSDWRFLRGYHQYA
1PYW	DRB1^*∗*^0101	XFVKQNAAALX
1KLG	DRB1^*∗*^0101	GELIGILNAAKVPAD
1KLU	DRB1^*∗*^0101	GELIGTLNAAKVPAD
2FSE	DRB1^*∗*^0101	AGFKGEQGPKGEPG
1SJH	DRB1^*∗*^0101	PEVIPMFSALSEG
1SJE	DRB1^*∗*^0101	PEVIPMFSALSEGATP
1T5W	DRB1^*∗*^0101	AAYSDQATPLLLSPR
1T5X	DRB1^*∗*^0101	AAYSDQATPLLLSPR
2IAN	DRB1^*∗*^0101	GELIGTLNAAKVPAD
2IAM	DRB1^*∗*^0101	GELIGILNAAKVPAD
2IPK	DRB1^*∗*^0101	XPKWVKQNTLKLAT
1FYT	DRB1^*∗*^0101	PKYVKQNTLKLAT
1R5I	DRB1^*∗*^0101	PKYVKQNTLKLAT
1HXY	DRB1^*∗*^0101	PKYVKQNTLKLAT
1JWM	DRB1^*∗*^0101	PKYVKQNTLKLAT
1JWS	DRB1^*∗*^0101	PKYVKQNTLKLAT
1JWU	DRB1^*∗*^0101	PKYVKQNTLKLAT
1LO5	DRB1^*∗*^0101	PKYVKQNTLKLAT
2ICW	DRB1^*∗*^0101	PKYVKQNTLKLAT
2OJE	DRB1^*∗*^0101	PKYVKQNTLKLAT
2G9H	DRB1^*∗*^0101	PKYVKQNTLKLAT
1A6A	DRB1^*∗*^0301	PVSKMRMATPLLMQA
1J8H	DRB1^*∗*^0401	PKYVKQNTLKLAT
2SEB	DRB1^*∗*^0401	AYMRADAAAGGA
1BX2	DRB1^*∗*^1501	ENPVVHFFKNIVTPR
1YMM	DRB1^*∗*^1501	ENPVVHFFKNIVTPRGGSGGGGG
1FV1	DRB5^*∗*^0101	NPVVHFFKNIVTPRTPPPSQ
1H15	DRB5^*∗*^0101	GGVYHFVKKHVHES
1ZGL	DRB5^*∗*^0101	VHFFKNIVTPRTPGG
4E41	DRB1^*∗*^0101	GELIGILNAAKVPAD
1DLH	DRB1^*∗*^0101	PKYVKQNTLKLAT
1KG0	DRB1^*∗*^0101	PKYVKQNTLKLAT
3L6F	DRB1^*∗*^0101	APPAYEKLSAEQSPP
3PDO	DRB1^*∗*^0101	KPVSKMRMATPLLMQALPM
3PGD	DRB1^*∗*^0101	KMRMATPLLMQALPM
3S4S	DRB1^*∗*^0101	PKYVKQNTLKLAT
3S5L	DRB1^*∗*^0101	PKYVKQNTLKLAT
1HQR	DRB5^*∗*^0101	VHFFKNIVTPRTP

**Table 2:** 30 HLA-complexes binding pockets.

PDB ID	Pocket 1	Pocket 2	Pocket 3	Pocket 4	Pocket 5	Pocket 6	Pocket 7	Pocket 8	Pocket 9
1AQD	82N 85V 86G	77T 78Y 81H 82N	78Y	13F 74A 78Y	13F 71R	11L	47Y 61W 67L 70Q 71R	60Y 61W	9W 57D 61W

1PYW	82N 85V 86G 89F	77T 78Y 81H 82N	78Y	13F 70Q 71R 74A 78Y	13F 71R	11L	11L 28E 61W 71R	60Y 61W	57D 61W

1KLG	82N 85V	78Y 81H 82N	78Y	13F 71R 78Y	13F 71R	11L	61W	60Y 61W	57D 61W

2FSE	82N 85V 86G 89F	77T 78Y 82N		13F 28E 70Q 71R 74A 78Y	13F 71R	71R	28E 47Y 61W 67L 71R	61W	57D

1KLU	82N 85V	78Y 81H 82N		13F 71R 78Y	13F 71R	11L	61W	60Y 61W	57D 61W

1SJH	82N	78Y 81H 82N		13F 26L 70Q 71R 74A 78Y	71R	11L	61W	60Y 61W	57D 61W

1SJE	82N	78Y 81H 82N	78Y	13F 26L 70Q 71R 74A 78Y	71R	11L	61W	60Y 61W	57D 60Y 61W

1T5W	82N 86G 89F	78Y 81H 82N	78Y	13F 70Q 71R 74A 78Y	13F 71R	11L	61W 71R	60Y 61W	9W 57D 61W

1T5X	82N 86G 89F	78Y 81H 82N	78Y	13F 70Q 71R 74A 78Y	71R	11L	61W 71R	61W	57D 61W

2IAN	82N 85V	78Y 81H 82N	78Y	13F 70Q 74A 78Y	13F 70Q 71R	11L	61W 71R	61W	57D 61W

2IPK	82N 85V 86G 89F	77T 78Y 81H 82N		13F 70Q 71R 74A 78Y	71R	11L	47Y 61W 67L 71R	60Y 61W	9W 57D 61W

1FYT	82N 85V 86G 89F	78Y 81H 82N	78Y	13F 70Q 71R 74A 78Y	13F 71R	11L	28E 47Y 61W 67L 71R	60Y 61W	9W 57D 61W

1R5I	82N 85V 86G 89F	77T 78Y 81H 82N	78Y	13F 70Q 71R 74A 78Y	70Q 71R	11L	47Y 61W 67L 71R	61W	9W 57D 61W

1HXY	82N 85V 86G 89F	78Y 81H 82N		13F 70Q 71R 74A 78Y	71R	11L	28E 47Y 61W 67L 71R	60Y 61W	9W 57D 61W

1JWM	82N 85V 86G 89F	78Y 81H 82N	78Y	13F 70Q 71R 74A 78Y	71R	11L	28E 47Y 61W 67L 71R	61W	57D 61W

1JWS	82N 85V 86G 89F	78Y 81H 82N	78Y	13F 70Q 71R 74A 78Y	13F 71R	11L	47Y 61W 67L 71R	61W	9W 57D 61W

1JWU	82N 85V 86G 89F	78Y 81H 82N	78Y	13F 70Q 71R 74A 78Y	13F 71R	11L	28E 47Y 61W 67L 71R	61W	9W 57D 61W

1LO5	82N 85V 86G 89F	78Y 81H 82N	78Y	13F 70Q 78Y	13F 71R	11L	47Y 61W 67L 71R	61W	9W 57D 60Y 61W

2ICW	82N 85V 86G 89F	78Y 81H 82N	78Y	13F 70Q 71R 74A 78Y	13F 71R	11L	28E 47Y 61W 67L 71R	61W	9W 57D 61W

2OJE	82N 85V 86G	77T 78Y 81H 82N	78Y	13F 70Q 71R 74A 78Y	70Q 71R	11L	28E 47Y 61W 67L 71R	61W	9W 57D 61W

2G9H	82N 85V 86G 89F	77T 78Y 81H 82N	78Y	13F 70Q 71R 74A 78Y	71R	11L 13F	28E 47Y 61W 67L 71R	60Y 61W	9W 57D 61W

2IAM	82N	78Y 81H 82N	78Y	13F 70Q 71R 74A 78Y	70Q 71R	11L	61W 67L 71R	60Y 61W	57D 61W

1A6A	82N 85V 86V	77T 78Y 81H 82N	78Y	13S 26Y 74R 78Y	71K 74R	11S 30Y	30Y 47F 61W 67L 71K	61W	9E 30Y 57D 61W

1J8H	82N 85V 86G 89F	77T 78Y 81H 82N	78Y	13H 26F 28D 70Q 74A 78Y	13H 70Q 71K	11V 13H 30Y	30Y 47Y 61W 67L	60Y 61W	37Y 57D 61W

2SEB	82N	77T 78Y 81H 82N		13H 26F 71K 78Y	13H 71K	30Y	30Y 47Y 61W	60Y 61W	61W

1BX2	82N 85V	77T 78Y 81H 82N	78Y	13H 26F 28D 70Q 74A 78Y	70Q	13R			57D 60Y 61W

1YMM	82N	77T 78Y 81H 82N	78Y	13R 26F 28D 70Q 74A 78Y	70Q	13R	61W 67I	61W	57D 61W

1FV1	82N 85V 86G 89F	78Y 81H 82N	78Y	13Y 71R 78Y	71R	13Y	61W 67L 71K	61W	57D

1H15	82N 89F	77T 78Y 81H 82N	78Y	13Y 71R 78Y	71R	11D 13Y 30D	61W		57D 60Y

1ZGL	82N 85V 89F	77T 78Y 81H 82N		13Y 26F 71R 78Y	13Y	13Y 28H 61W 71R	61W		57D 60Y 61W

**Table 3:** Important positions at the binding core for MHC II molecules.

Pocket	Important positions
Pocket 1	82 85 86 89
Pocket 2	77 78 81 82
Pocket 3	78
Pocket 4	11 13 26 28 70 71 74 78
Pocket 5	11 13 28 70 71 74
Pocket 6	11 13 28 70 71 74
Pocket 7	11 28 30 47 61 67 70 71
Pocket 8	60 61
Pocket 9	9 30 37 57 60 61

**Table 4:** Comparison of our binding prediction with other approaches. The 5th column is the result of our method, and 6th to 8th columns are results of TEPITOPE, MultiRTA, and NetMHCIIpan. The bold cell means one error.

PDB ID	Allele	Peptide	Core	Ours	TEPITOPE	MultiRTA	NetMHCIIpan-2.0
1AQD	DRB1^*∗*^0101	VGSDWRFLRGYHQYA	WRFLRGYHQ	WRFLRGYHQ	WRFLRGYHQ	WRFLRGYHQ	WRFLRGYHQ
1PYW	DRB1^*∗*^0101	XFVKQNAAALX	FVKQNAAAL	FVKQNAAAL	FVKQNAAAL	FVKQNAAAL	FVKQNAAAL
1KLG	DRB1^*∗*^0101	GELIGILNAAKVPAD	IGILNAAKV	IGILNAAKV	IGILNAAKV	IGILNAAKV	**LIGILNAAK**
2FSE	DRB1^*∗*^0101	GELIGTLNAAKVPAD	IGTLNAAKV	IGTLNAAKV	IGTLNAAKV	IGTLNAAKV	IGTLNAAKV
1KLU	DRB1^*∗*^0101	AGFKGEQGPKGEPG	FKGEQGPKG	FKGEQGPKG	FKGEQGPKG	FKGEQGPKG	FKGEQGPKG
1SJH	DRB1^*∗*^0101	PEVIPMFSALSEG	VIPMFSALS	VIPMFSALS	VIPMFSALS	VIPMFSALS	VIPMFSALS
1SJE	DRB1^*∗*^0101	PEVIPMFSALSEGATP	VIPMFSALS	VIPMFSALS	VIPMFSALS	VIPMFSALS	VIPMFSALS
1T5W	DRB1^*∗*^0101	AAYSDQATPLLLSPR	YSDQATPLL	**SDQATPLLL**	YSDQATPLL	**SDQATPLLL**	YSDQATPLL
1T5X	DRB1^*∗*^0101	AAYSDQATPLLLSPR	YSDQATPLL	**SDQATPLLL**	YSDQATPLL	**SDQATPLLL**	YSDQATPLL
2IAN	DRB1^*∗*^0101	GELIGTLNAAKVPAD	IGTLNAAKV	IGTLNAAKV	IGTLNAAKV	IGTLNAAKV	IGTLNAAKV
2IPK	DRB1^*∗*^0101	GELIGILNAAKVPAD	IGILNAAKV	IGILNAAKV	IGILNAAKV	IGILNAAKV	**LIGILNAAK**
1FYT	DRB1^*∗*^0101	XPKWVKQNTLKLAT	WVKQNTLKL	WVKQNTLKL	WVKQNTLKL	WVKQNTLKL	WVKQNTLKL
1R5I	DRB1^*∗*^0101	PKYVKQNTLKLAT	YVKQNTLKL	YVKQNTLKL	YVKQNTLKL	YVKQNTLKL	YVKQNTLKL
1HXY	DRB1^*∗*^0101	PKYVKQNTLKLAT	YVKQNTLKL	YVKQNTLKL	YVKQNTLKL	YVKQNTLKL	YVKQNTLKL
1JWM	DRB1^*∗*^0101	PKYVKQNTLKLAT	YVKQNTLKL	YVKQNTLKL	YVKQNTLKL	YVKQNTLKL	YVKQNTLKL
1JWS	DRB1^*∗*^0101	PKYVKQNTLKLAT	YVKQNTLKL	YVKQNTLKL	YVKQNTLKL	YVKQNTLKL	YVKQNTLKL
1JWU	DRB1^*∗*^0101	PKYVKQNTLKLAT	YVKQNTLKL	YVKQNTLKL	YVKQNTLKL	YVKQNTLKL	YVKQNTLKL
1LO5	DRB1^*∗*^0101	PKYVKQNTLKLAT	YVKQNTLKL	YVKQNTLKL	YVKQNTLKL	YVKQNTLKL	YVKQNTLKL
2ICW	DRB1^*∗*^0101	PKYVKQNTLKLAT	YVKQNTLKL	YVKQNTLKL	YVKQNTLKL	YVKQNTLKL	YVKQNTLKL
2OJE	DRB1^*∗*^0101	PKYVKQNTLKLAT	YVKQNTLKL	YVKQNTLKL	YVKQNTLKL	YVKQNTLKL	YVKQNTLKL
2G9H	DRB1^*∗*^0101	PKYVKQNTLKLAT	YVKQNTLKL	YVKQNTLKL	YVKQNTLKL	YVKQNTLKL	YVKQNTLKL
2IAM	DRB1^*∗*^0101	PKYVKQNTLKLAT	YVKQNTLKL	YVKQNTLKL	YVKQNTLKL	YVKQNTLKL	YVKQNTLKL
1A6A	DRB1^*∗*^0301	PVSKMRMATPLLMQA	MRMATPLLM	MRMATPLLM	MRMATPLLM	MRMATPLLM	MRMATPLLM
1J8H	DRB1^*∗*^0401	PKYVKQNTLKLAT	YVKQNTLKL	YVKQNTLKL	YVKQNTLKL	YVKQNTLKL	YVKQNTLKL
2SEB	DRB1^*∗*^0401	AYMRADAAAGGA	MRADAAAGG	MRADAAAGG	MRADAAAGG	MRADAAAGG	**YMRADAAAG**
1BX2	DRB1^*∗*^1501	ENPVVHFFKNIVTPR	VHFFKNIVT	VHFFKNIVT	VHFFKNIVT	VHFFKNIVT	**VVHFFKNIV**
1YMM	DRB1^*∗*^1501	ENPVVHFFKNIVTPRGGSGGGGG	VHFFKNIVT	VHFFKNIVT	VHFFKNIVT	VHFFKNIVT	VHFFKNIVT
1FV1	DRB5^*∗*^0101	NPVVHFFKNIVTPRTPPPSQ	FKNIVTPRT	**KNIVTPRTP**	FKNIVTPRT	**VHFFKNIVT**	**FFKNIVTPR**
1H15	DRB5^*∗*^0101	GGVYHFVKKHVHES	YHFVKKHVH	YHFVKKHVH	YHFVKKHVH	YHFVKKHVH	YHFVKKHVH
1ZGL	DRB5^*∗*^0101	VHFFKNIVTPRTPGG	FKNIVTPRT	**KNIVTPRTP**	FKNIVTPRT	**VHFFKNIVT**	**FFKNIVTPR**

Results				4 errors	0 errors	4 errors	6 errors

**Table 5:** Other prediction results of nine MHC molecules. This table shows the prediction result of our method on 9 MHC molecules. The 5th column is the result. There is only one error result, which is shown using bold font.

PDB ID	Allele	Peptide	Core	Ours
4E41	DRB1^*∗*^0101	GELIGILNAAKVPAD	IGILNAAKV	IGILNAAKV
1DLH	DRB1^*∗*^0101	PKYVKQNTLKLAT	YVKQNTLKL	YVKQNTLKL
1KG0	DRB1^*∗*^0101	PKYVKQNTLKLAT	YVKQNTLKL	YVKQNTLKL
3L6F	DRB1^*∗*^0101	APPAYEKLSAEQSPP	YEKLSAEQS	YEKLSAEQS
3PDO	DRB1^*∗*^0101	KPVSKMRMATPLLMQALPM	MRMATPLLM	**KMRMATPLL**
3PGD	DRB1^*∗*^0101	KMRMATPLLMQALPM	MRMATPLLM	MRMATPLLM
3S4S	DRB1^*∗*^0101	PKYVKQNTLKLAT	YVKQNTLKL	YVKQNTLKL
3S5L	DRB1^*∗*^0101	PKYVKQNTLKLAT	YVKQNTLKL	YVKQNTLKL
1HQR	DRB5^*∗*^0101	VHFFKNIVTPRTP	FKNIVTPRT	FKNIVTPRT

Results				1 error
