# Supplementary material for: A Novel Peptide Binding Prediction Approach for HLA-DR Molecule Based on Sequence and Structural Information
Source: Biomed Res Int. 2016 May 31;2016:3832176. doi: 10.1155/2016/3832176 (PMC4906198; doi:10.1155/2016/3832176)
Supplement: Supplementary file 1 — Using different functions to combine sequence similarity and structural similarity, these are the predicted results with the value of alpha ranging from 1 to 5. [file 3832176.f1.pdf]

Supplementary Materials

Table S1 Predicted result by Score1 function

PDB	Allel	Peptide	Core	α =1	α =2	α =3	α =4	α =5
1AQ	DRB1	VGSDWRFLRGY	WRFL	WRFL	WRFL	WRFL	WRFL	WRFL
1PY	DRB1	XFVKQNAAALX	FVKQ	FVKQ	FVKQ	FVKQ	FVKQ	FVKQ
1KL	DRB1	GELIGILNAAKV	IGILN	IGILN	IGILN	IGILN	IGILN	IGILN
2FSE	DRB1	GELIGTLNAAKV	IGTLN	IGTLN	IGTLN	IGTLN	IGTLN	IGTLN
1KL	DRB1	AGFKGEQGPKG	FKGE	FKGE	FKGE	FKGE	FKGE	FKGE
1SJH	DRB1	PEVIPMFSALSE	VIPM	VIPM	VIPM	VIPM	VIPM	VIPM
1SJE	DRB1	PEVIPMFSALSE	VIPM	VIPM	VIPM	VIPM	VIPM	VIPM
1T5	DRB1	AAYSDQATPLLL	YSDQ	SDQA	SDQA	SDQA	SDQA	SDQA
1T5	DRB1	AAYSDQATPLLL	YSDQ	SDQA	SDQA	SDQA	SDQA	SDQA
2IA	DRB1	GELIGTLNAAKV	IGTLN	IGTLN	IGTLN	IGTLN	IGTLN	IGTLN
2IPK	DRB1	GELIGILNAAKV	IGILN	IGILN	IGILN	IGILN	IGILN	IGILN
1FYT	DRB1	XPKWVKQNTLK	WVK	WVK	WVK	WVK	WVK	WVK
1R5I	DRB1	PKYVKQNTLKLA	YVKQ	YVKQ	YVKQ	YVKQ	YVKQ	YVKQ
1HX	DRB1	PKYVKQNTLKLA	YVKQ	YVKQ	YVKQ	YVKQ	YVKQ	YVKQ
1JW	DRB1	PKYVKQNTLKLA	YVKQ	YVKQ	YVKQ	YVKQ	YVKQ	YVKQ
1JW	DRB1	PKYVKQNTLKLA	YVKQ	YVKQ	YVKQ	YVKQ	YVKQ	YVKQ
1JW	DRB1	PKYVKQNTLKLA	YVKQ	YVKQ	YVKQ	YVKQ	YVKQ	YVKQ
1LO	DRB1	PKYVKQNTLKLA	YVKQ	YVKQ	YVKQ	YVKQ	YVKQ	YVKQ
2IC	DRB1	PKYVKQNTLKLA	YVKQ	YVKQ	YVKQ	YVKQ	YVKQ	YVKQ
2OJ	DRB1	PKYVKQNTLKLA	YVKQ	YVKQ	YVKQ	YVKQ	YVKQ	YVKQ
2G9	DRB1	PKYVKQNTLKLA	YVKQ	YVKQ	YVKQ	YVKQ	YVKQ	YVKQ
2IA	DRB1	PKYVKQNTLKLA	YVKQ	YVKQ	YVKQ	YVKQ	YVKQ	YVKQ
1A6	DRB1	PVSKMRMATPL	MRM	MRM	MRM	MRM	MRM	MRM
1J8	DRB1	PKYVKQNTLKLA	YVKQ	YVKQ	YVKQ	YVKQ	YVKQ	YVKQ
2SE	DRB1	AYMRADAAAG	MRA	MRA	MRA	MRA	MRA	MRA
1BX	DRB1	ENPVVHFFKNIV	VHFF	VHFF	VHFF	VHFF	VHFF	VHFF
1YM	DRB1	ENPVVHFFKNIV	VHFF	VHFF	VHFF	VHFF	VHFF	VHFF
1FV	DRB5	NPVVHFFKNIVT	FKNIV	KNIVT	KNIVT	KNIVT	KNIVT	KNIVT
1H1	DRB5	GGVYHFVKKHV	YHFV	YHFV	YHFV	YHFV	YHFV	YHFV
1ZG	DRB5	VHFFKNIVTPRT	FKNIV	KNIVT	KNIVT	KNIVT	KNIVT	KNIVT
Resu				4	4	4	4	4

Table S2 Predicted result by Score2 function

PDB	Allele	Peptide	Core	α =1	α =2	α =3	α =4	α =5
1AQ	DRB1	VGSDWRFLRGY	WRFL	VGSD	WRFL	WRFL	WRFL	WRFL
1PY	DRB1	XFVKQNAAALX	FVKQ	FVKQ	FVKQ	FVKQ	FVKQ	FVKQ
1KL	DRB1	GELIGILNAAKVP	IGILN	LIGIL	LIGILN	LIGILN	LIGILN	LIGILN
2FSE	DRB1	GELIGTLNAAKV	IGTLN	LIGTL	LIGTL	LIGTL	LIGTL	LIGTL
1KL	DRB1	AGFKGEQGPKG	FKGE	FKGE	FKGE	FKGE	FKGE	FKGE
1SJH	DRB1	PEVIPMFSALSE	VIPM	VIPM	VIPM	VIPM	VIPM	VIPM
1SJE	DRB1	PEVIPMFSALSE	VIPM	VIPM	VIPM	VIPM	VIPM	VIPM
1T5	DRB1	AAYSDQATPLLL	YSDQ	AYSD	AYSD	AYSD	AYSD	AYSD
1T5X	DRB1	AAYSDQATPLLL	YSDQ	AYSD	AYSD	AYSD	AYSD	AYSD
2IAN	DRB1	GELIGTLNAAKV	IGTLN	LIGTL	LIGTL	LIGTL	LIGTL	LIGTL
2IPK	DRB1	GELIGILNAAKVP	IGILN	LIGIL	LIGILN	LIGILN	LIGILN	LIGILN
1FYT	DRB1	XPKWVKQNTLK	WVK	WVK	WVK	WVK	WVK	WVK
1R5I	DRB1	PKYVKQNTLKLA	YVKQ	YVKQ	YVKQ	YVKQ	YVKQ	YVKQ
1HX	DRB1	PKYVKQNTLKLA	YVKQ	YVKQ	YVKQ	YVKQ	YVKQ	YVKQ
1JW	DRB1	PKYVKQNTLKLA	YVKQ	YVKQ	YVKQ	YVKQ	YVKQ	YVKQ
1JW	DRB1	PKYVKQNTLKLA	YVKQ	YVKQ	YVKQ	YVKQ	YVKQ	YVKQ
1JW	DRB1	PKYVKQNTLKLA	YVKQ	YVKQ	YVKQ	YVKQ	YVKQ	YVKQ
1LO	DRB1	PKYVKQNTLKLA	YVKQ	YVKQ	YVKQ	YVKQ	YVKQ	YVKQ
2IC	DRB1	PKYVKQNTLKLA	YVKQ	YVKQ	YVKQ	YVKQ	YVKQ	YVKQ
2OJE	DRB1	PKYVKQNTLKLA	YVKQ	YVKQ	YVKQ	YVKQ	YVKQ	YVKQ
2G9	DRB1	PKYVKQNTLKLA	YVKQ	YVKQ	YVKQ	YVKQ	YVKQ	YVKQ
2IA	DRB1	PKYVKQNTLKLA	YVKQ	YVKQ	YVKQ	YVKQ	YVKQ	YVKQ
1A6	DRB1	PVSKMRMATPL	MRM	MRM	MRM	MRM	MRM	MRM
1J8H	DRB1	PKYVKQNTLKLA	YVKQ	KQNT	YVKQ	YVKQ	YVKQ	YVKQ
2SEB	DRB1	AYMRADAAAGG	MRA	MRA	MRAD	MRAD	MRAD	MRAD
1BX	DRB1	ENPVVHFFKNIV	VHFF	VHFF	VHFFK	VHFFK	VHFFK	VHFFK
1YM	DRB1	ENPVVHFFKNIV	VHFF	VHFF	VHFFK	VHFFK	VHFFK	VHFFK
1FV	DRB5	NPVVHFFKNIVT	FKNIV	KNIV	KNIVT	KNIVT	KNIVT	KNIVT
1H1	DRB5	GGVYHFVKKHV	YHFV	YHFV	YHFV	YHFV	YHFV	YHFV
1ZGL	DRB5	VHFFKNIVTPRTP	FKNIV	KNIV	KNIVT	KNIVT	KNIVT	KNIVT
Resu				10	8	8	8	8

Table S3 Predicted result by Score3 function

PDB ID	Allele	Peptide	Core	α =1	α =2	α =3	α =4	α =5
1AQD	DRB1*0101	VGSDWRFLRGYHQYA	WRFLRGYHQ	VGSDWRFLR	VGSDWRFLR	WRFLRGYHQ	WRFLRGYHQ	WRFLRGYHQ
1PYW	DRB1*0101	XFVKQNAAALX	FVKQNAAAL	FVKQNAAAL	FVKQNAAAL	FVKQNAAAL	FVKQNAAAL	FVKQNAAAL
1KLG	DRB1*0101	GELIGILNAAKVPAD	IGILNAAKV	LIGILNAAK	LIGILNAAK	LIGILNAAK	LIGILNAAK	LIGILNAAK
2FSE	DRB1*0101	GELIGTLNAAKVPAD	IGTLNAAKV	LIGTLNAAK	LIGTLNAAK	LIGTLNAAK	LIGTLNAAK	LIGTLNAAK
1KLU	DRB1*0101	AGFKGEQGPKGEPG	FKGEQGPKG	FKGEQGPKG	FKGEQGPKG	FKGEQGPKG	FKGEQGPKG	FKGEQGPKG
1SJH	DRB1*0101	PEVIPMFSALSEG	VIPMFSALS	VIPMFSALS	VIPMFSALS	VIPMFSALS	VIPMFSALS	VIPMFSALS
1SJE	DRB1*0101	PEVIPMFSALSEGATP	VIPMFSALS	VIPMFSALS	VIPMFSALS	VIPMFSALS	VIPMFSALS	VIPMFSALS
1T5W	DRB1*0101	AAYSDQATPLLLSPR	YSDQATPLL	AYSDQATPL	AYSDQATPL	AYSDQATPL	AYSDQATPL	AYSDQATPL
1T5X	DRB1*0101	AAYSDQATPLLLSPR	YSDQATPLL	AYSDQATPL	AYSDQATPL	AYSDQATPL	AYSDQATPL	AYSDQATPL
2IAN	DRB1*0101	GELIGTLNAAKVPAD	IGTLNAAKV	LIGTLNAAK	LIGTLNAAK	LIGTLNAAK	LIGTLNAAK	LIGTLNAAK
2IPK	DRB1*0101	GELIGILNAAKVPAD	IGILNAAKV	LIGILNAAK	LIGILNAAK	LIGILNAAK	LIGILNAAK	LIGILNAAK
1FYT	DRB1*0101	XPKWVKQNTLKLAT	WVKQNTLKL	WVKQNTLKL	WVKQNTLKL	WVKQNTLKL	WVKQNTLKL	WVKQNTLKL
1R5I	DRB1*0101	PKYVKQNTLKLAT	YVKQNTLKL	YVKQNTLKL	YVKQNTLKL	YVKQNTLKL	YVKQNTLKL	YVKQNTLKL
1HXY	DRB1*0101	PKYVKQNTLKLAT	YVKQNTLKL	YVKQNTLKL	YVKQNTLKL	YVKQNTLKL	YVKQNTLKL	YVKQNTLKL
1JWM	DRB1*0101	PKYVKQNTLKLAT	YVKQNTLKL	YVKQNTLKL	YVKQNTLKL	YVKQNTLKL	YVKQNTLKL	YVKQNTLKL
1JWS	DRB1*0101	PKYVKQNTLKLAT	YVKQNTLKL	YVKQNTLKL	YVKQNTLKL	YVKQNTLKL	YVKQNTLKL	YVKQNTLKL
1JWU	DRB1*0101	PKYVKQNTLKLAT	YVKQNTLKL	YVKQNTLKL	YVKQNTLKL	YVKQNTLKL	YVKQNTLKL	YVKQNTLKL
1LO5	DRB1*0101	PKYVKQNTLKLAT	YVKQNTLKL	YVKQNTLKL	YVKQNTLKL	YVKQNTLKL	YVKQNTLKL	YVKQNTLKL
2ICW	DRB1*0101	PKYVKQNTLKLAT	YVKQNTLKL	YVKQNTLKL	YVKQNTLKL	YVKQNTLKL	YVKQNTLKL	YVKQNTLKL
2OJE	DRB1*0101	PKYVKQNTLKLAT	YVKQNTLKL	YVKQNTLKL	YVKQNTLKL	YVKQNTLKL	YVKQNTLKL	YVKQNTLKL
2G9H	DRB1*0101	PKYVKQNTLKLAT	YVKQNTLKL	YVKQNTLKL	YVKQNTLKL	YVKQNTLKL	YVKQNTLKL	YVKQNTLKL
2IAM	DRB1*0101	PKYVKQNTLKLAT	YVKQNTLKL	YVKQNTLKL	YVKQNTLKL	YVKQNTLKL	YVKQNTLKL	YVKQNTLKL
1A6A	DRB1*0301	PVSKMRMATPLLMQA	MRMATPLLM	MRMATPLLM	MRMATPLLM	MRMATPLLM	MRMATPLLM	MRMATPLLM
1J8H	DRB1*0401	PKYVKQNTLKLAT	YVKQNTLKL	YVKQNTLKT	YVKQNTLKT	YVKQNTLKT	YVKQNTLKT	YVKQNTLKT
2SEB	DRB1*0401	AYMRADAAAGGA	MRADAAAGG	MRADAAAGG	MRADAAAGG	MRADAAAGG	MRADAAAGG	MRADAAAGG
1BX2	DRB1*1501	ENPVVHFFKNIVTPR	VHFFKNIVT	VHFFKNIVT	VHFFKNIVT	VHFFKNIVT	VHFFKNIVT	VHFFKNIVT
1YMM	DRB1*1501	ENPVVHFFKNIVTPRGGSGGGGG	VHFFKNIVT	VHFFKNIVT	VHFFKNIVT	VHFFKNIVT	VHFFKNIVT	VHFFKNIVT
1FV1	DRB5*0101	NPVVHFFKNIVTPRTPPPSQ	FKNIVTPRT	KNIVTPRTP	KNIVTPRTP	KNIVTPRTP	KNIVTPRTP	KNIVTPRTP
1H15	DRB5*0101	GGVYHFVKKHVHES	YHFVKKHVH	YHFVKKHVH	YHFVKKHVH	YHFVKKHVH	YHFVKKHVH	YHFVKKHVH
1ZGL	DRB5*0101	VHFFKNIVTPRTPGG	FKNIVTPRT	KNIVTPRTP	KNIVTPRTP	KNIVTPRTP	KNIVTPRTP	KNIVTPRTP
Results				9 errors	9 errors	8 errors	8 errors	8 errors
